# Community-based antibiotic access and use in six low-income and middle-income countries: a mixed-method approach

**DOI:** 10.1016/S2214-109X(21)00024-3

**Published:** 2021-03-10

**Authors:** Nga T T Do, Huong T L Vu, Chuc T K Nguyen, Sureeporn Punpuing, Wasif Ali Khan, Margaret Gyapong, Kwaku Poku Asante, Khatia Munguambe, F Xavier Gómez-Olivé, Johannes John-Langba, Toan K Tran, Malee Sunpuwan, Esperanca Sevene, Hanh H Nguyen, Phuc D Ho, Mohammad Abdul Matin, Sabeena Ahmed, Mohammad Mahbubul Karim, Olga Cambaco, Samuel Afari-Asiedu, Ellen Boamah-Kaali, Martha Ali Abdulai, John Williams, Sabina Asiamah, Georgina Amankwah, Mary Pomaa Agyekum, Fezile Wagner, Proochista Ariana, Betuel Sigauque, Stephen Tollman, H Rogier van Doorn, Osman Sankoh, John Kinsman, Heiman F L Wertheim

**Affiliations:** a Oxford University Clinical Research Unit, Hanoi, Vietnam; b Department of Family Medicine, Hanoi Medical University, Hanoi, Vietnam; c Institute for Population and Social Research, Mahidol University, Nakhonpathom, Thailand; d International Centre for Diarrhoeal Disease Research, Dhaka, Bangladesh; e Institute of Health Research, University of Health and Allied Sciences, Ho, Ghana; f Kintampo Health Research Centre, Kintampo, Ghana; g Manhiça Health Research Centre, Manhiça, Mozambique; h Faculty of Medicine, Eduardo Mondlane University, Maputo, Mozambique; i MRC–Wits Rural Public Health and Health Transitions Research Unit (Agincourt), University of the Witwatersrand, Johannesburg, South Africa; j School of Public Health, University of the Witwatersrand, Johannesburg, South Africa; k School of Applied Human Sciences, University of Kwazulu-Natal, Durban, South Africa; l Institute of Mathematics, Vietnam Academy of Science and Technology, Hanoi, Vietnam; m Dodowa Health Research Centre, Dodowa, Ghana; n Nuffied Department of Clinical Medicine, University of Oxford, Oxford, UK; o Statistics Sierra Leone, Freetown, Sierra Leone; p University Secretariat, Njala University, Njala, Sierra Leone; q Heidelberg Institute for Global Health, University of Heidelberg Medical School, Heidelberg, Germany; r Department of Epidemiology and Global Health, Umeå University, Umeå, Sweden; s Department of Medical Microbiology and Radboudumc Center for Infectious Diseases, Radboudumc, Nijmegen, The Netherlands

## Abstract

**Background:**

Antimicrobial misuse is common in low-income and middle-income countries (LMICs), and this practice is a driver of antibiotic resistance. We compared community-based antibiotic access and use practices across communities in LMICs to identify contextually specific targets for interventions to improve antibiotic use practices.

**Methods:**

We did quantitative and qualitative assessments of antibiotic access and use in six LMICs across Africa (Mozambique, Ghana, and South Africa) and Asia (Bangladesh, Vietnam, and Thailand) over a 2·5-year study period (July 1, 2016–Dec 31, 2018). We did quantitative assessments of community antibiotic access and use through supplier mapping, customer exit interviews, and household surveys. These quantitative assessments were triangulated with qualitative drug supplier and consumer interviews and discussions.

**Findings:**

Vietnam and Bangladesh had the largest proportions of non-licensed antibiotic dispensing points. For mild illness, drug stores were the most common point of contact when seeking antibiotics in most countries, except South Africa and Mozambique, where public facilities were most common. Self-medication with antibiotics was found to be widespread in Vietnam (55·2% of antibiotics dispensed without prescription), Bangladesh (45·7%), and Ghana (36·1%), but less so in Mozambique (8·0%), South Africa (1·2%), and Thailand (3·9%). Self-medication was considered to be less time consuming, cheaper, and overall, more convenient than accessing them through health-care facilities. Factors determining where treatment was sought often involved relevant policies, trust in the supplier and the drug, disease severity, and whether the antibiotic was intended for a child. Confusion regarding how to identify oral antibiotics was revealed in both Africa and Asia.

**Interpretation:**

Contextual complexities and differences between countries with different incomes, policy frameworks, and cultural norms were revealed. These contextual differences render a single strategy inadequate and instead necessitate context-tailored, integrated intervention packages to improve antibiotic use in LMICs as part of global efforts to combat antibiotic resistance.

**Funding:**

Wellcome Trust and Volkswagen Foundation.

## Introduction

Antibiotic resistance has created an urge for global efforts to facilitate appropriate use of antibiotics. Global reports on antibiotic resistance have shown that studies are needed in low-income and middle-income countries (LMICs), where local data are scarce and resistance is widespread.^1^, ^2^ In a report^3^ quantifying antibiotic exposure in children younger than 5 years in eight African LMICs between 2006 and 2016, antibiotics were prescribed to 80·5% (15 480 of 19 231) of children diagnosed with respiratory illness, with a large proportion of these prescriptions deemed unnecessary. Between 2000 and 2010, worldwide antibiotic consumption for human use increased by 35%, particularly in LMICs, with high levels of antibiotic use both inside and outside the formal health-care system.^4^ Consumption of broad-spectrum antibiotics also increased in LMICs, suggesting increased access and a response to an increase in resistance.^5^ Reducing unnecessary use of antibiotics would benefit patients in terms of preventing side-effects and in reinforcing global efforts to control antibiotic resistance.

A systematic review^6^ covering 34 studies on factors associated with self-medication of antimicrobials in LMICs showed that 39% of respondents self-medicate. Pharmacies, drug stores, and leftover or borrowed drugs were identified as main sources of self-medication. The key determinants included severity of illness, economic status, past successful antibiotic use and educational level. Another review^7^ covering 15 studies investigating factors affecting self-medication of antimicrobials in LMICs found a broad range in the prevalence of self-medication, from 8% to 93%. Sociocultural determinants of health associated with the structure and conditions of the health system, including accessibility, affordability, health-care facilities conditions, and health-seeking behaviour, were found to be the main factors affecting self-medication in studied LMICs. A systematic review of 85 studies,^8^ 59 of which were done in LMICs, found that more than 60% of antibiotics were dispensed without a prescription in 49 (83%) of the studies. However, there is a considerable heterogeneity in these systematic reviews due to an absence of standardised criteria for collection of survey data. These studies only focused on either the supply or the demand side, were limited to adults, used little data from African settings, and their methods often risked recall bias.^9^ Moreover, there is a scarcity of comprehensive and contextual studies considering local complexities to guide future research and support the development of evidence-based interventions in promoting appropriate antibiotic use across different LMICs.

**Research in context**

**Evidence before this study**

Community-based antibiotic access and use has received less attention than prescription behaviours in formal health-care settings. We have little understanding of antibiotic use in the community, which is considered a key driver of antibiotic resistance. We searched PubMed and Google Scholar for articles published between Jan 1, 2000, and Jan 12, 2021, and we restricted our search to articles published in English that reported factors associated with antibiotic use in low-income and middle-income countries (LMICs) using the search terms “antibiotic”, “self-medication”, and “low- and middle-income countries”. We found three systematic reviews covering 85 studies on antibiotic dispensing without a prescription worldwide, of which 59 were published in LMICs, 34 studied factors affecting self-medication with antimicrobials in LMICs, and 15 investigated factors affecting self-medication with antibiotics in LMICs. These studies revealed a high prevalence of self-medication. Accessibility, affordability, health-care facility conditions, and health-seeking behaviour were found to be the main determinants of self-medication in studied LMICs. However, there is considerable heterogeneity in the reviewed studies due to an absence of standardised criteria for survey data collection. The reviewed studies were limited to self-reporting on questionnaires, focused on either the supply or the demand side in households, and provided few insights regarding community practices to guide future interventions. Furthermore, most studies focused on Asia with little research on misuse of antibiotics in Africa, and there is a scarcity of comprehensive and contextual studies considering local complexities to guide future research and support the development of evidence-based interventions in promoting appropriate antibiotic use across different LMICs.

**Added value of this study**

To fill these research gaps, we used a comparative mixed-method approach to compare community-based antibiotic access and use practices across communities in six LMICs in Asia (Bangladesh, Thailand, and Vietnam) and Africa (Mozambique, Ghana, and South Africa) to allow for comparison by national income status and identify supply and demand drivers. Within the existing INDEPTH surveillance network, our study was powered to identify the contextual complexities and differences between countries with different income levels, policy frameworks, and cultural norms. The gained insight in antibiotic practices in different LMICs could inform the design of context-adjusted models for interventions to effectively address the usage of antibiotics and contain antibiotic resistance in these resource-constrained settings.

**Implications of all the available evidence**

Our study found common themes reported in previous studies in LMICs, but also revealed the diversity and complexity of locally specific sociocultural determinants affecting antibiotic access and use among studied communities in six Asian and African LMICs. The identified factors will serve as targets for development of context-tailored interventions to effectively address the misuse of antibiotics and contain antibiotic resistance in LMICs.

To fill these research gaps, we aimed to use a comparative approach to assess access and use practices across communities in six LMICs in Asia (Bangladesh, Thailand, and Vietnam) and Africa (Mozambique, Ghana, and South Africa) to allow for comparison by national income status and identify key drivers through both qualitative and quantitative measures.

## Methods

### Study design and participants

Operating from the existing INDEPTH surveillance network, a global network of health and demographic surveillance sites, this study (the ABACUS project) was done in seven communities in six LMICs in Africa (Kintampo [rural] and Dodowa [suburban], Ghana; Manhiça [rural], Mozambique; and Agincourt [rural], South Africa) and Asia (Matlab [rural], Bangladesh; Kanchanaburi [urban and suburban], Thailand; and Filabavi [rural], Vietnam). For each study site, all possible purchase or dispensing points for antibiotics (antibiotic suppliers) were included, comprising any formal or informal antibiotic supplier from public hospital pharmacies to street vendors. Country scans for the health-care system and antibiotic legal framework are provided in the appendix (pp 13–17). Other details on rationale for study sites and sample selection are also provided in the appendix (pp 1–2) and were published previously.^10^

We used a mixed-methods approach with in-depth interviews and focus group discussions among drug suppliers and consumers, household surveys, and exit interviews among customers who purchased antibiotics. The study was done between July 1, 2016, and Dec 31, 2018, in four parts. Part 1 consisted of mapping of suppliers and inventories, and part 2 consisted of preparatory in-depth interviews and focus group discussions with suppliers (16 in-depth interviews per site) and community members (16 in-depth interviews and six focus group discussions per site). Part 3 involved quantitative longitudinal household surveys of Health and Demographic Surveillance Sites in two rounds, during a 1-year period (1100 households per site with 5–10% loss to follow-up). This number was adjusted for South Africa (600 households per site) and Mozambique (650 households per site) due to higher costs of fieldwork. Simultaneously, community antibiotic supply was quantified through standardised customer exit interviews at four timepoints over a 1-year study period (Aug 1, 2017–July 31, 2018) and synchronised across sites. In part 4, to explain any potential discrepancies between results from the household survey and the customer exit survey, further explanatory in-depth interviews and focus group discussions with selected groups (five in-depth interviews and two focus group discussions each site) were done.^10^

Antibiotic suppliers aged 18 years or older were eligible for qualitative interviews (in-depth interviews and focus group discussions). Ideally, each interviewee was affiliated with a unique antibiotic supplier, but if fewer than 16 eligible antibiotic suppliers could be identified at each site, a maximum of two employees from the same supplier could participate in the in-depth interviews but not in the same focus group discussion. Similarly, community members aged 18 years or older who were willing to share their experience and attitudes toward medicines were eligible to participate in qualitative interviews (in-depth interviews and focus group discussions). Participants from different age categories and of different sexes were involved to achieve a more informative view. Different targeted populations included men and women aged 18–60 years, men and women older than 60 years, and mothers who cared for children younger than 5 years, due to a higher frequency of health-care seeking. Two participants from the same household were avoided. Community members who participated in in-depth interviews were excluded from focus group discussion and vice versa. For the household surveys, households that already participated in an ongoing health and demographic surveillance system and had previously provided consent were eligible to participate in this survey. An adult household representative (18 years or older) needed to provide additional written informed consent before the household could be included in the survey. Other details on inclusion criteria and rationale for participant selection were published previously.^10^

The study was approved by the Oxford University Tropical Research Ethics Committee (31-15), and the local ethical committees of each participating study site.^10^ Written informed consent was obtained from participants (suppliers and community members) in all data collection activities (mapping, household survey, customer exit interview, in-depth interviews, and focus group discussions).

### Quantitative analysis

Data were collected using tablets and stored using a web-based application: Research Electronic Data Capture. Data were extracted, cleaned, and analysed in R version 3.3.3. Descriptive data were described as mean (SD), median (IQR), and proportion (95% CI). The analysis and presentation of the quantitative data followed the STROBE guidelines for reporting observational studies.^11^ We used a generalised linear model to examine the factors associated with family use of antibiotics and estimated the adjusted odds ratios (ORs) and their 95% CIs. Community antibiotic consumption was estimated in defined daily doses supplied according to WHO's 2019 AWaRe (Access, Watch, and Reserve) classification.^12^ The factors associated with the supply of Watch antibiotics were examined using multivariable logistic regression analysis.

For each of the studied communities, through observed metrics, we aimed to map all formal and informal suppliers of antibiotics, inventory antibiotic resources, and determine community antibiotic exposure and the appropriateness of the antibiotics supplied. Through reported experiences, we aimed to assess demographic, socioeconomic, and cultural characteristics, the accessibility of health care, and frequency of antibiotic intake and characterise the practices involved in antibiotic demand, access, and use (eg, indication, source, knowledge, and expectations).

### Qualitative analysis

A thematic analytical approach was used on the English language summaries of each in-depth interview and focus group discussion for each study site. Because all the interviews and discussions were done in local languages, these summaries were made to facilitate centralised comparative analysis. The summaries were produced when the transcript was coded following a standardised format. The analysis was based on a-priori themes from the various topics that had been covered during the in-depth interviews and focus group discussions, with additional themes and subthemes emerging inductively during the reading and analysis process. Verification of the data and themes was done by members of the research team with skills in qualitative analysis (NTTD, MG, JJ-L, PA, and JK). This verification involved validating and assessing the trustworthiness of the qualitative data using the four constructs of credibility, transferability, dependability, and conformability.^10^ The analysis was facilitated by QRS International NVivo (version 12) software. The analysis and presentation of the qualitative data followed COREQ guidelines for reporting qualitative research.^13^

Through reported experiences, we aimed to investigate health-care concepts and behaviours and socioeconomic and cultural characteristics associated with practices of antibiotic access and use in each studied context.

### Role of the funding source

The funder of the study had no role in study design, data collection, data analysis, data interpretation, or writing of the report.

## Results

Supplier mapping showed that Asian sites had a higher density of antibiotic suppliers than African sites, with the highest density found in urban Thailand (5 per 1000 inhabitants) and the lowest in rural South Africa (1 per 10 000 inhabitants; table 1). In Asia, more than 90% of mapped suppliers were private providers: 467 (93%) of 502 in Vietnam, 278 (92·4%) of 301 in Bangladesh, and 278 (95%) of 293 in Thailand. Ghana had a similarly high percentage of private providers (98 [80%] of 122), whereas Mozambique (18 [38%] of 47) and South Africa (3 [20%] of 15) had lower percentages. Vietnam (325 [65%] of 502) and Bangladesh (156 [52%] of 301) had the highest proportion of providers operating without legal authorisation (non-licensed providers) compared with other countries (10 [21%] of 47 in Mozambique, five [4%] of 122 in Ghana, 0 [0%] of 2913 Thailand, and 0 [0%] of 15 in South Africa; table 1).

**Table 1:** Key demographic indicators and antibiotic access at surveyed sites

		**Low-income countries**		**Lower middle-income countries**		**Upper middle-income countries**	
		Bangladesh	Mozambique	Vietnam	Ghana	Thailand	South Africa
Site name		Matlab	Manhica	Filabavi	Dodowa; Kintampo	Kanchanaburi	Agincourt
Site population		225 000	165 346	262 000	117 341; 142 977	59 966	120 000
Under-5 mortality per 1000 livebirths (year)		37·4 (2011)	76·1 (2014)	8·4 (2011)	32·8 (2011); 62·6 (2011)	8·5 (2009)	48·0 (2009)
Number of antibiotics suppliers		301	47	502	122*	293	15
	Non-licensed, n (%)	156 (52%)	10 (21%)	325 (65%)	5 (4%)*	0	0
	Private, n (%)	278 (92%)	18 (38%)	467 (93%)	97 (80%)*	278 (95%)	3 (20%)

* Mean data for both sites are presented. Separate data for each site are provided in the appendix (p 11).

8214 exit interviews were done with customers who purchased antibiotics at 140 suppliers (appendix p 3). The proportions of antibiotics dispensed without prescription were greater in the low-income and lower middle-income countries (1168 [36·1%] of 3237 for Ghana, 844 [45·7%] of 1859 for Bangladesh, and 773 [55·2%] of 1399 for Vietnam) compared with upper middle-income countries (18 [3·9%] of 462 for Thailand and five [1·2%] of 418 for South Africa). An exception was Mozambique, which was a low-income country with a relatively small proportion of antibiotics purchased without prescription (67 [8·0%] of 839). Across sites, the average proportion of antibiotics dispensed without prescription was 2875 (35·0%) of 8214 interviewed customers.

6190 households with 25 274 individuals were assessed for their practices regarding antibiotic access and use (appendix pp 4–5). Across sites, 2290 (37·0%) households reported self-treatment with antibiotics. For mild illness, drug stores in Vietnam (2630 [72·5%] of 3627) and Bangladesh (4024 [91·0%] of 4424) and public health-care facilities in Mozambique (797 [80·9%] of 985) and South Africa (2303 [95·1%] of 2422) were the predominant first point of contact for household members (table 2). Drug stores were also the most common point of contact when seeking antibiotics for children with mild illness in all countries, except for Vietnam, where the drug store was a more common first point of contact when purchasing antibiotics for adults (1991 [79·7%] of 2498) than when purchasing for children (638 [56·6%] of 1127).

**Table 2:** Care-seeking behaviours among households included in the household survey—first point of contact when household members were unwell

	**Bangladesh**	**Mozambique**	**Vietnam**	**Ghana**	**Thailand**	**South Africa**
**In mild illness**						
N*	4424	985	3627	9742	3707	2422
Drug store	4024 (91·0%)†	57 (5·8%)	2630 (72·5%)†	4967 (51·0%)†	1548 (41·8%)†	10 (0·4%)
Private facility	36 (0·8%)	35 (3·6%)	241 (6·6%)	978 (10·0%)	1134 (30·6%)	90 (3·7%)
Public facility	327 (7·4%)	797 (80·9%)†	411 (11·3%)‡	2840 (29·2%)	920 (24·8%)	2303 (95·1%)†
Other	37 (0·8%)	96 (9·7%)	347 (9·5%)	957 (9·8%)	105 (2·8%)	19 (0·8%)
**In severe illness**						
N*	4419	984	3629	9746	3706	2422
Drug store	28 (0·6%)	9 (0·9%)	5 (0·1%)	218 (2·2%)	2 (0·1%)	8 (0·3%)
Private facility	1672 (37·8%)	29 (2·9%)	841 (23·2%)	1714 (17·6%)	590 (15·9%)	126 (5·2%)
Public facility	2716 (61·5%)†	907 (92·2%)†	2690 (74·1%)†‡	7111 (73·0%)†	3093 (83·5%)†	2275 (93·9%)†
Other	3 (0·1%)	39 (4·0%)	93 (2·6%)	703 (7·2%)	21 (0·6%)	13 (0·5%)

Data are n (%), unless otherwise specified.

* The number of responses for the most common first point of contact for the individual household members in each site.

† Most common first point of contact.

‡ Includes communal health stations.

Qualitative analysis showed that, although public health-care facilities were predominantly chosen for more severe illness in all countries, drug stores were the first point of contact for treatment of mild symptoms in most settings, except Mozambique and South Africa. In in-depth interviews and focus group discussions with community members, most people in South Africa and Mozambique reported that they relied on treatment from primary health-care facilities, including antibiotics, which were provided for free. However, in Mozambique, when drugs were unavailable at health-care facilities, people could acquire them from private pharmacies where antibiotics are more costly.

From in-depth interviews and focus group discussions, factors determining where treatment was sought for a household often involved policy-associated determinants and a balance between trust in the supplier and the drug, convenience of access, disease severity, and whether or not the consumer was a child (panel). Where regulations were more enforced, community members were aware that it was not permitted to buy antibiotics directly from the pharmacy without prescription (South Africa). Although antibiotics were highly regulated by national policies at authorised suppliers in Thailand, they were still illegally distributed by unauthorised dispensers. In in-depth interviews with community members, the main concern regarding illegally distributed antibiotics was so-called Yaa Chud (poly-pharmaceutical packs containing antibiotics), which are widely available in community-based grocery stores. In low-income and lower middle-income countries, where the regulation was less likely to be enforced, participants indicated that self-treatment was less time consuming, cheaper, and overall, more convenient than the public health services, corroborating what we found in the quantitative results.

Panel **Demand-side factors associated with antibiotic access and use shared by studied communities**

**Care seeking behaviour**

*Policy-associated behaviour*

“Here in South Africa, you cannot get antibiotics without the referral from the doctor.” (focus group discussion 1, woman, 30 years, South Africa)

“If I want to buy Yaa Chud [poly-pharmaceutical packs containing antibiotics], I just walk to the grocery shop nearby.” (in-depth interview 13, woman, 68 years, Thailand)

*Perception of health-care facilities*

“It's not difficult to get to the hospital, the biggest problem is the delay to be seen, and sometimes the lack of pills at the hospital, what compels us to buy at the private pharmacy where [antibiotics] are so expensive and we do not have money to pay.” (focus group discussion 4, man, 30–45 years, Mozambique)

“The treatment is for free but people are hesitant to use it. Instead they want something that they will pay for.” (in-depth interview 2, man, 64 years, South Africa)

“I would like to take treatment in government hospital, but I don't get proper service there. They don't do their assigned duty, neglect the patients.” (focus group discussion 6, man, 30–45 years, Bangladesh)

*Trust in supplier*

“When I have mild symptoms, I always go to the drug store first. I don't even know the name [of the medications] and their effect because I trust them completely. I only go to the hospital when I get more severe illness.” (focus group discussion 7, woman with children younger than 5 years, 25 years, Vietnam)

*Convenience of access*

“I prefer buying medicine from a nearby drug store than going to the hospital. At the pharmacy, they ask the symptoms before they give the drugs and they are very effective.” (focus group discussion 4, woman, 32 years, Ghana)

“Most people don't like going to the hospital. When they visit the hospital, they will sit there for a very long time before they are attended to, so patients may end up dying with the illness they took to the hospital. So they will move straight to the drug store to buy medicines to cure their illness.” (focus group discussion 6, woman, 29 years, Ghana)

*Perceived disease severity*

“Our family members did not like to go see a doctor, except when we got a serious sickness. If someone has a fever or cough, we buy Yaa Chud [non-prescribed poly-pharmaceutical packs containing antibiotics] from the drug store.” (in-depth interview 6, woman with children younger than 5 years, 36 years, Thailand)

“I only go to the hospital when I get more severe illness.” (focus group discussion 1, woman with children younger than 5 years, 38 years, Vietnam)

*Children are a priority*

“If children are sick, it is a must to bring them to hospital.” (focus group discussion 5, woman, 35 years, Vietnam)

**Perception of antibiotics**

*Misperception of antibiotics' function*

“Antibiotics are powerful drugs, which are used for serious illnesses. When paracetamol does not work for cough and cold then antibiotics should be used.” (focus group discussion 7, woman with children younger than 5 years, 21 years, Vietnam)

“Recently, febrile illness could not be cured without antibiotics, and I have to spend thousands BDT [Bangladesh currency] every month for buying antibiotics”. (focus group discussion 1, man, 50 years, Bangladesh)

“People are not dying like in the past; it means they are using antibiotics in a good way.” (focus group discussion 8, woman, 21–30 years, South Africa)

*Confusion of antibiotic with anti-inflammatory*

“We don't know them by name. They have two colours.” (focus group discussion 4, man, 42 years, Mozambique)

“There are different types of antibiotics. We have the red and yellow [amoxicillin] that is used for stomach pains and wounds. There is a different type that is red it is also used for waist pains.” (focus group discussion 2, woman, 27 years, Ghana) “I know some [antibiotics] such as ‘Amox’ [amoxicillin], ‘Tiffy’ [paracetamol], ‘Panadol’ [paracetamol], ‘anpha choay’ [anpha-chymotripsin].” (focus group discussion 1, woman with child younger than 5 years, 21 years, Vietnam)

*Limited knowledge of antibiotic resistance*

“I find that illness resolves after consuming antibiotics very quickly, but I think it creates another disease in our body and gives rise to side-effects. People are not aware of this properly.” (focus group discussion 2, woman, 40 years, Bangladesh)

“Recently, many people in my village have had common cold or flu due to the changing season. The disease has been more severe and difficult to treat as it has become resistant to flu medicines. Higher doses of antibiotics were, therefore, needed to recover.” (focus group discussion 8, man, 65 years, Vietnam)

**Practice**

*Self-treatment linked to previous experience*

“If you have previously used an antibiotic, you just go and buy one from the drug store. Recently, I bought some antibiotics (Amoxicillin), because I knew what I wanted [so] I just went to the drug store and asked for it.” (in-depth interview 10, woman, 20 years, Ghana)^19^

*Taking incomplete courses*

“I won't deny it, sometimes I also do that [laughing], I take pills and when I see I am better, especially if the pills are bitter, I stop taking them, I quit.” (focus group discussion 1, woman, 18–30 years, Mozambique)

“They prescribe drug for me to buy some medicine, but I couldn't buy all the medicine, so I bought some of the medicine, and 2 weeks later, I bought the rest of the medicine that time all my feet got swollen.” (in-depth interview 16, woman, 21 years, Ghana)

*Shared with others or save for future use*

“I share medicines with my neighbours and I usually ask them for Napa [paracetamol], peridon [domperidone], and antibiotics for dysentery.” (in-depth interview 1, man, 40 years, Bangladesh)

“We keep them so that we can use them again when we get ill.” (focus group discussion 4, woman, 18–26 years, South Africa)

**Figure 1:**
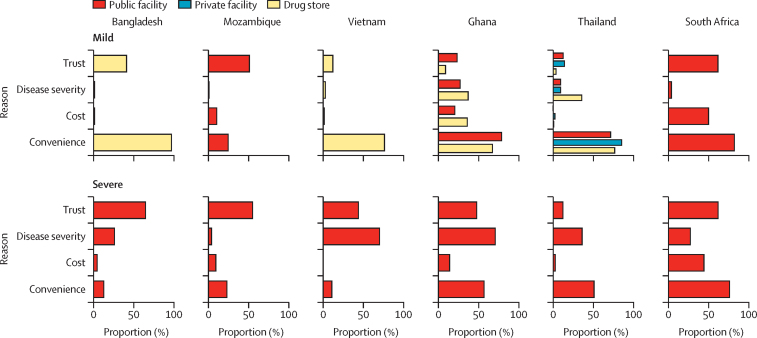
Reasons for choosing the selected first point of contact in case of mild and severe illness for household members For mild illness in Ghana and Thailand, more than one option was selected in similar proportions to those shown.

In household surveys, most respondents said they chose where to access antibiotics for mild illness on the basis of how convenient the location was to access, except Mozambique, where trust was the most common reason for choosing a public health-care facility (figure 1). For severe illness, trust was among the most common reasons for choosing a public facility, except in Thailand. Convenience was the most common reason for South Africa and Thailand, whereas disease severity was the most common for Vietnam and Ghana.

In Bangladesh, participants in focus group discussions shared that public health services at the district level or lower are free of charge but are considered low standard, with few types of antibiotics available. Therefore, community members mainly rely on nearby drug sellers (those commonly perceived as doctors by local people) for mild conditions. In Ghana, the main and first approached sources for antibiotics were licensed chemical sellers (over-the-counter medicine sellers who are not allowed by regulations to sell antibiotics other than co-trimoxazole) and drug peddlers (street vendors who sell drugs illegally). Findings from focus group discussions suggested that delays in receiving care at hospitals encouraged people to seek medicine from the licensed chemical sellers rather than from public health-care providers (panel). This common reason was also reported in Vietnam, where there is a high density of private and non-licensed antibiotic suppliers.

The largest proportion of households reporting use of antibiotics in the previous month in the first household survey was in Bangladesh (498 [49·4%] of 1009), followed by Vietnam (416 [45·0%] of 925), Ghana (728 [37·4%] of 1947), Thailand (294 [27·9%] of 1053), Mozambique (161 [25·2%] of 639), and South Africa (63 [10·2%] of 616; table 3). These proportions were similar in the second round. However, the proportions of antibiotic use without prescription in the second round were substantially decreased in Mozambique and increased in Ghana compared with the first round (appendix p 8). Households without health insurance coverage were less likely to have used antibiotics (adjusted OR 0·82, 95% CI 0·70–0·96). Having access to drug stores for health care (1·26, 1·07–1·47) and for information (1·17, 1·03–1·33) increased household antibiotic use. In the repeated survey, apart from site and number of household members, reported antibiotic use in the first round (1·38, 1·19–1·59; p<0·0001) and getting information from drug stores (1·29, 1·03–1·62; p=0·031) was significantly associated with the reported use in the second round (appendix p 12). The proportion of households self-treating with antibiotics varied from zero (0·0%) of 72 in South Africa to 448 (65·3%) of 686 in Bangladesh in the first survey. In the second survey, this proportion was increased for Ghana (532 [70·6%] of 754; p<0·0001) but reduced for Mozambique (two [1·4%] of 139; p<0·0001), and it remained similar for other countries (appendix p 8).

**Table 3:** Family use of antibiotics in the previous month in the first survey round and associated factors

	**Use, n/N (%)**	**No use, n/N (%)**	Adjusted OR*(95% CI)	**p value**
**Site**				
Bangladesh	498/1009 (49·4%)	511/1009 (50·6%)	1 (ref)	..
Mozambique	161/639 (25·2%)	478/639 (74·8%)	0·59 (0·46–0·75)	<0·0001
Vietnam	416/925 (45·0%)	509/925 (55·0%)	0·71 (0·56–0·89)	0·0038
Ghana (Kintampo)	465/1100 (42·3%)	635/1100 (57·7%)	0·73 (0·57–0·94)	0·014
Ghana (Dodowa)	263/847 (31·1%)	584/847 (68·9%)	0·50 (0·39–0·65)	<0·0001
Thailand	294/1053 (27·9%)	759/1053 (72·1%)	0·50 (0·39–0·64)	<0·0001
South Africa	63/616 (10·2%)	553/616 (89·8%)	0·21 (0·15–0·29)	<0·0001
**Number of people in the household**				
1–4	1050/3553 (29·6%)	2503/3553 (70·4%)	1 (ref)	..
>4	1108/2624 (42·2%)	1516/2624 (57·8%)	1·00 (1·00–1·00)	0·38
**Sex of respondent**				
Male	592/1853 (31·9%)	1261/1853 (68·1%)	1 (ref)	..
Female	1559/4314 (36·1%)	2755/4314 (63·9%)	1·09 (0·99–1·20)	0·082
**Highest education level in the family**				
Secondary school or higher	1706/4727 (36·1%)	30 201/4727 (63·9%)	1 (ref)	..
Primary school or less	454/1462 (31·1%)	1008/1462 (68·9%)	0·95 (0·84–1·06)	0·34
**Health-care coverage**				
Free health insurance	963/2937 (32·8%)	1974/2937 (67·2%)	1 (ref)	..
Paid health insurance	388/1094 (35·5%)	706/1094 (64·5%)	0·98 (0·84–1·13)	0·76
Out-of-pocket payment	809/2158 (37·5%)	1349/2158 (62·5%)	0·82 (0·70–0·96)	0·013
**Health-care options available to the household**				
Public hospitals	965/3208 (30·1%)	2243/3208 (69·9%)	1 (ref)	..
Private hospitals	632/1782 (35·5%)	1150/1782 (64·5%)	1·13 (0·97–1·32)	0·11
Drug stores	419/822 (51·0%)	403/822 (49·0%)	1·26 (1·07–1·47)	0·0054
Other options	144/377 (38·2%)	233/377 (61·8%)	1·16 (0·86–1·57)	0·33
**Drug supplier household members attended**				
Public hospitals	1011/3311 (30·5%)	2300/3311 (69·5%)	1 (ref)	..
Private hospitals	473/1336 (35·4%)	863/1336 (64·6%)	0·96 (0·83–1·13)	0·64
Drug stores	487/1086 (44·8%)	599/1086 (55·2%)	0·96 (0·82–1·13)	0·64
Other suppliers	189/456 (41·4%)	267/456 (58·6%)	1·21 (0·91–1·61)	0·19
**Factors determines choices of drug supplier**				
Convenience	612/1782 (34·3%)	1170/1782 (65·7%)	1 (ref)	..
Trust	846/2320 (36·5%)	1474/2320 (63·5%)	1·01 (0·90–1·14)	0·81
Cost	491/1501 (32·7%)	1010/1501 (67·3%)	1·02 (0·89–1·16)	0·79
Other factors	211/586 (36·0%)	375/586 (64·0%)	0·95 (0·77–1·17)	0·64
**Source of information**				
Health-care facilities	495/1817 (27·2%)	1322/1817 (72·8%)	1 (ref)	..
Drug stores	798/1969 (40·5%)	1171/1969 (59·5%)	1·17 (1·03–1·33)	0·016
Print materials	163/540 (30·2%)	377/540 (69·8%)	1·23 (1·02–1·50)	0·034
Other sources	704/1863 (37·8%)	1159/1863 (62·2%)	1·07 (0·94–1·23)	0·32
**Knowledge about antibiotics, number of correct answers out of 4**				
0	226/1073 (21·1%)	847/1073 (78·9%)	1 (ref)	..
1	746/1979 (37·7%)	1233/1979 (62·3%)	1·43 (1·23–1·67)	<0·0001
2	340/830 (41·0%)	490/830 (59·0%)	1·66 (1·38–1·99)	<0·0001
3	353/951 (37·1%)	598/951 (62·9%)	1·65 (1·37–1·98)	<0·0001
4	482/1315 (36·7%)	833/1315 (63·3%)	1·54 (1·28–1·85)	<0·0001

Other comparisons across the two sites in Ghana are presented in the appendix (appendix p 11). OR=odds ratio.

* ORs are adjusted using a generalised linear model that include all the listed variables.

**Figure 2:**
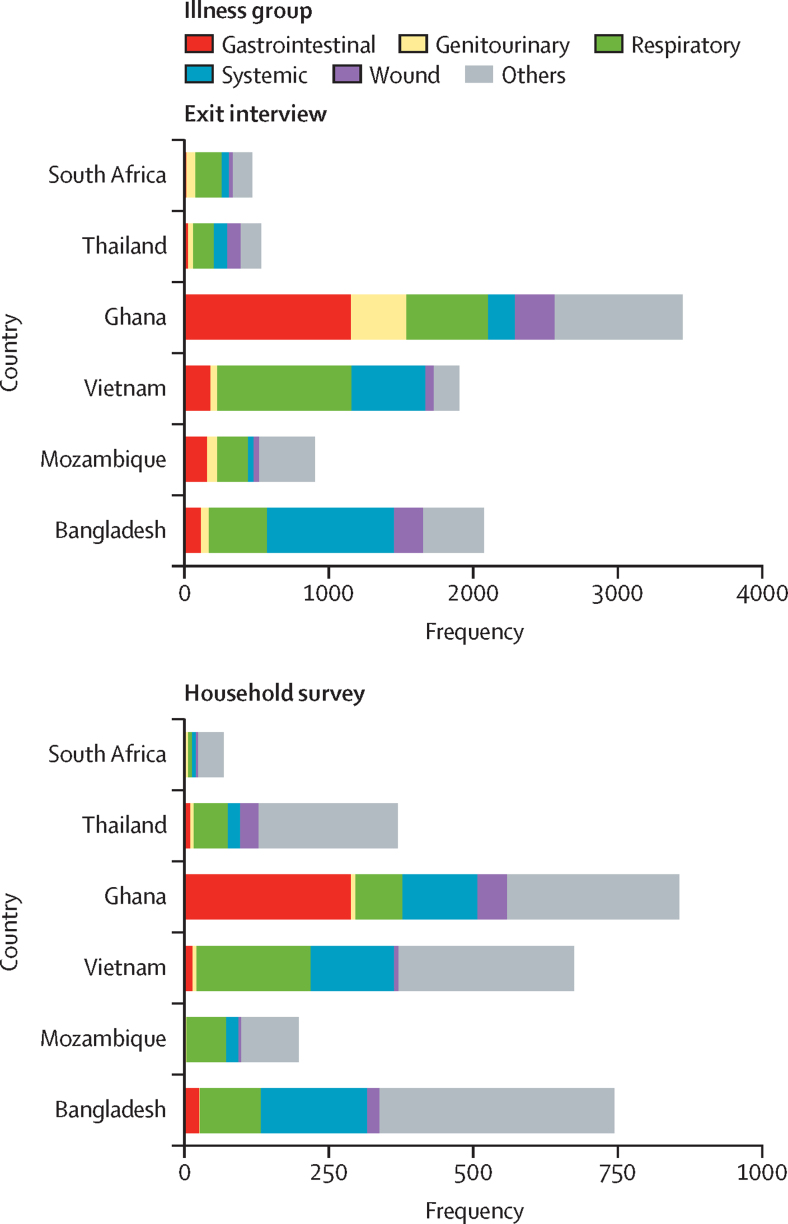
Reported indications for antibiotics obtained at drug suppliers through exit interviews and reported antibiotic use by household members in the past month, obtained through household surveys Bars represent counts of individuals with specific indication, counts are not mutually exclusive except for the “Others” category. Respiratory category includes throat, cough, nose, chest pain, and dyspnoea symptoms. Systemic category includes headache, non-localised pain, weakness, and fever symptoms. Genitourinary category includes sexually transmitted infections, gynaecological, male genital, and urinary tract infection.

Misperceptions about antibiotics were common among respondents in the household survey, especially confusion about what antibiotics do and when they should be taken (appendix p 6). Antibiotics were bought for different indications across the surveyed countries. Indications commonly reported in exit interviews and household surveys included respiratory (cough), systemic (fever), and gastrointestinal symptoms (in Ghana only; figure 2). More antibiotics were purchased for upper-respiratory symptoms in Vietnam (767 [53%] of 1457 purchases for cough and 675 [46%] for throat) and for fever in Bangladesh (874 [46%] of 1897) than in other countries in exit interviews. However, obtaining antibiotics for gastrointestinal symptoms was more common in Ghana (1253 [35%] of 3600 purchases overall, 1046 [41%] of 2521 in Kintampo and 207 [19%] of 1079 in Dodowa) than in the other sites. More participants in Thailand reported purchasing antibiotics for skin wounds (100 [19%] of 525 purchases) than in other countries.

Across sites, both suppliers (in-depth interviews) and consumers (household surveys) could not always distinguish antibiotics from other commonly sold medicines like painkillers (eg, tramadol) or antipyretics. Antibiotics were often identified by colour and shape, such as red and green capsules or pink elongated pills. The wide variety in names and appearances contributed to potential misidentification (appendix p 7). Medications dispensed as capsules were regularly considered to be antibiotics, despite the fact that this is neither exclusive nor universal for antibiotics.

Across all sites, it was commonly reported in focus group discussions that antibiotics were used for similar conditions as found in the quantitative results. Participants in Bangladesh, Mozambique, and Vietnam thought antibiotics were medications for most diseases, including infections that were probably non-bacterial. In African sites, malaria and sexually transmitted infections were more commonly indicated conditions. Regardless of perceived treatable conditions, antibiotics were considered to be powerful and life-saving medicine across both continents (panel).

Access-group antibiotics were predominant in almost all sites (ranging from 3664 [50·7%] of 7222 defined daily doses in Vietnam to 1764 [84·6%] of 2052 defined daily doses in South Africa), except Bangladesh, where the Watch-group antibiotics accounted for 7414 (74·6%) of 9934 defined daily doses (appendix pp 9–10). No Reserve-group antibiotics were supplied at any of the study sites.

Qualitative findings from in-depth interviews and focus group discussions with community members showed that antibiotic use without prescription was a common practice in almost all sites except South Africa. It was commonly reported to be based on previous treatment experience.

Community members were not aware that antibiotic courses should be completed and that antibiotics can be harmful to their body. In low-income countries, lack of money forces community members to buy incomplete courses of treatment. Even when people could afford a full course, they still bought a few pills to see if they worked (ie, pills were sold in small numbers or doses). In South Africa where patients received full courses prescribed by health-care workers, patients did not always complete their courses, because they did not feel the need to take more pills after they recovered. Unused medicines were shared with others or saved for future use in almost all sites but could be returned and refunded in Bangladesh, Ghana, and Vietnam (panel).

## Discussion

Our results showed that a large proportion of antibiotics were acquired without a prescription across study sites in low-income and lower middle-income countries. 2875 (35·0%) of the 8214 customers interviewed across all six countries had purchased antibiotics without a prescription. This finding aligned with those from the household survey, in which 2290 (37·0%) of 6190 households reported self-medication with antibiotics. Despite the legal requirements to dispense antibiotics only with prescription, non-compliance was found to be widespread in the low-income and lower middle-income countries, but not in the two upper middle-income countries. This non-compliance could be a focus of future interventions, which will require context-adjusted models for optimal effect. In rural South Africa, for example, little health care was available and a clear governance structure that made antibiotics accessible only through prescription was adhered to, limiting access. Additional factors also limited access in South Africa, including ability to reach and to pay.^14^ One exception was rural Mozambique, a low-income country with a relatively small proportion of antibiotics purchased without prescription (67 [8·0%] of 839), caused not by better enforcement but rather by limited access, as well as availability of free health care provided by clinical studies to participating patients.^15^, ^16^ Our findings confirm the common practice of accessing antibiotics without prescriptions in LMICs that has previously been reported.^6^, ^17^, ^18^ A common theme was self-treatment being less time consuming, cheaper, and overall more convenient than using public health services.

Although prescription-only regulations for antibiotics might not be practical for many LMICs,^19^, ^20^, ^21^ easy access to antibiotics, particularly to broad-spectrum antibiotics in rural settings in Asia, raises concerns about high levels of antibiotic use outside the formal health-care system and will require interventions that target both the supply and demand sides to reduce inappropriate antibiotic use.^22^, ^23^ Watch-group antibiotics (access to which should be restricted in the community) formed larger proportions of antibiotic sales in Asian sites compared with African sites. Frequent use of Watch antibiotics was mainly associated with use for children in all Asian sites and purchasing without a prescription in Ghana. No Reserve-group antibiotics were dispensed at any of the study sites. As reflected in our findings, drug stores were the predominant first point of contact in case of mild illness owing to convenience, time, distance, and cost, whereas trust was the rationale for those who chose public services. Even in an environment with more heavily enforced policy (Thailand), self-medication with unknown medicines that include antibiotics (Yaa Chud) is a relatively common practice among older adults and migrant workers.^24^ Therefore, the potential conflict of interest, in which drug sellers are in the position of offering health recommendations to either benefit their customers or their own financial profits, needs to be addressed. Strengthening capacity of community pharmacists and raising their awareness about the role of the pharmacist in health-care systems to promote prudent antibiotic use in the community are therefore crucial.^25^, ^26^, ^27^ The role of the public health system, where patients chose to go because they trusted the provider during severe illness, or out of convenience in case of mild illness, should also be recognised. Considering the larger population that the public system serves and that they normally have no incentive to prescribe and sell antibiotics, interventions targeting these facilities remain crucial. Providing quality care by educational interventions, rapid diagnostic tests, increasing insurance coverage, and community sensitisation could lead to better use of public health-care services at the grassroots level.^28^, ^29^, ^30^

Confusion regarding how to identify oral antibiotics was revealed in both Africa and Asia. This issue needs to be considered before other issues of demand are addressed.^31^ Community members should be able to distinguish an antibiotic from other commonly sold medicines, such as painkillers or antipyretics, regardless of where they obtain the medicine. Clear labelling of antibiotics empowers community members to be conscious about use of antibiotics and enables them to put any future communication campaign about better use of antibiotics into practice. At present, they are reliant on information provided by drug sellers, which was often inadequate or entirely absent. Lessons can be learnt from the Red Line public awareness campaign in India, which made it mandatory to display a red vertical band on packaging of prescription-only drugs to improve identification and awareness of the dangers of antibiotic misuse.^32^ This campaign aligns with the global strategy of making communities more resilient in dealing with public health emergencies and the trend of involving patients more in treatment decisions.

Despite the richness of the data, this study has several limitations. First, the study was done in individual rural or urban and suburban communities in LMICs and is thus not fully representative of misuse of antibiotics in each country. Second, we might not have captured all antibiotic use, especially without prescriptions, by household members due to poor recall, self-perceived misuse of antibiotics, or lack of awareness that pills taken were antibiotics. Third, our stratified sampling resulted in an imbalanced proportion of supplier types owing to the small number of formal suppliers versus informal suppliers in some sites. Nevertheless, the collected information covers all types of suppliers that the community members are exposed to. Finally, we experienced challenges in approaching non-licensed antibiotics suppliers, which predominated in Asia, due to their refusal to participate in several study components. We might therefore have underestimated the proportion of inappropriate antibiotic use.

This study showed that antibiotic use differed markedly between study sites in LMICs and that access generally appeared to be more restricted in African than Asian locations. In Asian sites, antibiotics were widely available through a high density of both formal and informal antibiotic suppliers, and we found more dispensing without a prescription relative to African sites. The associated contextual factors will serve as targets for development of context-tailored, integrated intervention packages that target both the supply and demand side, to improve antibiotic use in these settings, and will contribute to global efforts to combat antibiotic resistance.

## Data sharing

Data may be made available according to data sharing policy of the local partners in the six participating countries upon request to the corresponding author.
