# Supplementary material for: Community-based antibiotic access and use in six low-income and middle-income countries: a mixed-method approach
Source: Lancet Glob Health. 2021 Mar 10;9(5):e610–9. doi: 10.1016/S2214-109X(21)00024-3 (PMC8050200; doi:10.1016/S2214-109X(21)00024-3)
Supplement: Supplementary appendix [file mmc1.pdf]

THE LANCET
Global Health

Supplementary appendix

This appendix formed part of the original submission and has been peer reviewed. We post it as supplied by the authors.

Supplement to: Do NTT, Vu HTL, Nguyen CTK, et al. Community-based antibiotic access and use in six low-income and middle-income countries: a mixed-method approach. *Lancet Glob Health* 2021; published online March 10. https://doi.org/10.1016/S2214-109X(21)00024-3.

Supplementary Table 1. Several key country indicators

	**LIC**		**MIC-L**		**MIC-U**	
	**Bangladesh**	**Mozambique**	**Vietnam**	**Ghana**	**Thailand**	**South Africa**
Total population (2013)	156,595,000	25,834,000	89,709,000	25,905,000	67,010,000	53,157,000
Proportion of population 0-14 years old (%, 2013)	30	46	23	39	18	30
Gross national income per capita (World Bank $, 2013)	1,010	610	1,740	1,770	5,340	7,410
Human development index * (2013)	0.56 medium	0.39 low	0.64 medium	0.57 medium	0.72 high	0.66 medium
% Rural population (2013)	67	68	68	47	52	36
Life expectancy at birth m/f (years, 2013)	70/71	49/51	71/80	60/62	71/78	55/59
Under 5 mortality per 1000 live births (latest)	41	87	24	78	13	44
Total expenditure on health per capita (Intl $, 2012)	32	40	111	100	264	593
Total expenditure on health as %	3.7	6.8	6.0	5.4	4.6	8.9

of GDP (2012)						
HIV prevalence adults (2013)	<0.1%	10.80%	0.40%	1.30%	1.10%	19.10%

Source: The World Bank, www.worldbank.org (accessed on May 15, 2015). *Human development index (HDI) is a composite index measuring average achievement in three basic dimensions of human development: a long and healthy life, knowledge and a decent standard of living (source: http://hdr.undp.org/en/content/human-development-index-hdi-table). Cut offs for low, medium, high and very high development are 0.55, 0.7 and 0.8 respectively.

Supplementary Table 2 – Respondents, by sexs, for the different data collection methods in ABACUS study

	Supplier IDIs (M:F)	Community IDIs (M:F)	Community FGD (M:F)	Exit interviews (M:F)	Household survey (M:F)	Total (M:F)
**Africa**						
Mozambique	10:6	3:13	12:12	340:499	186:452*	551:982
Ghana	15:17	25:7	48:48	1184:2053*	677:1266*	1949:3391
South Africa	11:6	5:12	20:23	120:298	183:429*	339:768
**Asia**						
Bangladesh	13:3	4:12	20:23	783:1076	164:845	984:1959
Vietnam	4:12	4:12	13:36	441:958*	279:645	486:1150
Thailand	6:10	11:5	18:22	138:324*	366:685	539:1046
**TOTAL**	**59:54**	**52:61**	**131:164**	**3006:5208**	**1855:4322**	**5103:9809**

M: Male; F: Female; IDI: In-depth Interview; FGD: Focus Group Discussion

*11 respondents in Ghana, 8 in Mozambique, 17 in Bangladesh, 4 in Vietnam, and 6 in Thailand with unspecified gender in exit interviews. 7 respondents in Ghana, 1 in South Africa, 1 in Mozambique, 3 in Thailand, and 1 in Vietnam with unspecified gender in household survey

Supplementary Table 3 – Characteristics of respondents and their households in household survey round 1 in six countries

**Characteristics**	**BD** (N=1009)	**MZ** (N=639)	**VN** (N=925)	**GN** (N=1950)	**THA** (N=1054)	**SA** (N=613)
Age (years), median (IQR)	44 (34-57)	44 (32-60)	52 (40-62)	41 (31-55)	55 (45-63)	40 (27-56)
Number of household members, median (IQR)	4 (3-5)	3 (1-6)	4 (3-5)	5 (3-7)	3 (2-5)	3 (2-5)
Main breadwinner's occupation, count (%)						
Farmer	235 (23.3)	286 (44.7)	293 (32.2)	874 (44.9)	89 (8.4)	9 (1.5)
Government staff	19 (1.9)	17 (2.7)	36 (4.0)	87 (4.5)	114 (10.8)	34 (5.6)
Manual worker	75 (7.4)	71 (11.1)	213 (23.4)	192 (9.9)	344 (32.6)	189 (30.9)
Housewife	110 (10.9)	19 (3.0)	0 (0)	14 (0.7)	2 (0.2)	7 (1.1)
Service	169 (16.8)	14 (2.2)	0 (0)	12 (0.6)	0 (0)	3 (0.5)
Business	193 (19.1)	6 (0.9)	0 (0)	241 (12.4)	111 (10.5)	20 (3.3)
Other	136(13.5)	171(26.7)	92 (10.1)	345 (17.7)	332(31.5)	118(19.3)
Not in the workforce	71 (7.0)	56 (8.7)	277 (30.4)	182 (9.3)	62 (5.9)	232 (37.9)
Healthcare cover						
Paid insurance	0 (0)	92 (14.4)	589 (63.7)	626 (32.2)	175 (16.6)	45 (7.3)
Free insurance	1 (0.1)	107 (16.7)	488 (52.8)	1021 (52.5)	749 (71.1)	569(93.0)
Out of pocket payment	1005(99.9)	607 (95.0)	706 (76.4)	1373 (70.7)	885 (84.0)	210 (34.3)

VN: Vietnam; BD: Bangladesh; THA: Thailand; MZ: Mozambique; GN: Ghana (Kintampo and Dodowa); SA: South Africa. N was based on the respondents who responded to the question on the most frequent means of traveling to the most frequently used healthcare provider for the household.

Supplementary Table 4 – Perception about antibiotics across sites

Characteristics	BD (N=1009)	MZ (N=639)	VN (N=925)	GN (N=1950)	THA (N=1054)	SA (N=613)
Having correct knowledge about antibiotics						
Which of the following types of medicine do you think is an antibiotic?*	202 (20.1)	58 (9.0)	779(84.5)	1070 (55.0)	575 (54.6)	253 (41.1)
What do you think antibiotics do? **	146 (14.5)	44 (6.9)	755(81.5)	735 (37.8)	537 (50.9)	242 (39.4)
When do you think should antibiotics be taken?#	112 (11.2)	25 (3.9)	708(76.5)	86 (4.4)	451 (42.8)	208 (33.8)
Which one pill do you think is an antibiotic?##	689 (68.4)	397(62.0)	844(91.5)	1890(97.1)	702 (66.6)	319 (52.0)

VN: Vietnam; BD: Bangladesh; THA: Thailand; MZ: Mozambique; GN: Ghana (Kintampo and Dodowa); SA: South Africa. N was based on the respondents who responded to the questions. *Following choices were provided: painkiller, drug against fatigue, drug against high blood pressure, drug against infection, other, don't know. ** Following choices were provided: decrease blood pressure, give energy, kill bacteria, stop pain, treat stomach ache, other, don't know. #Following choices were provided: bladder infection, muscle pain, weakness, stomach ache, other, don't know. ##Following choices were provided: photo of paracetamol, photo of non-steroid anti-inflammatory drug, photo of antibiotic, don't know.

Supplementary Table 5. Local term for antibiotic across sites

**Country**	**Local term for antibiotic**
Bangladesh	Antibiotics<br>*Joralo Oushudh* – Strong medicine
Mozambique	*Antibiótico* – Antibiotic<br>Red and Yellow caps<br>Capsules - medicines with rod shape<br>Medicines to treat infections and remove body pain
Vietnam	*Kháng sinh* - Antibiotic<br>*Con nhộng* - Capsule
Ghana	Abom Belt<br>*Tupaye* – Gun Powder<br>*Pa tsupa* - Sore medicine<br>Red-yellow
Thailand	*ยาฆ่าเชื้อ (Ya kha cheua)* - germicide<br>*ยาแก้อักเสบ (Ya kae aksep)* – anti-inflammatory / anti-infective<br>*ยาปฏิชีวนะ (Ya paṭichiwana*)-Antibiotics
South Africa	*Anthibhayotiki Maphilisi lama lwaka na switsongwatsongwana*<br>- Pills to fight microorganisms

Supplementary Table 6. Antibiotic practice in the previous month among households surveyed in the first survey and follow-up survey (6 months after the first survey)

Characteristic	Bangladesh	Mozambique	Vietnam	Ghana	Thailand	South Africa
**First survey**						
Antibiotic use*	498/1009 (49·4)	161/639 (25·2)	416/925 (45·0)	728/1947 (37·4)	294/1053 (27·9)	63/616 (10·2)
Without prescription**	448/686 (65·3)	68/243 (28·0)	231/510 (45·3)	363/1163 (31·2)	22/426 (5·2)	0/72 (0)
**Follow-up survey**						
Antibiotic use*	435/963 (45.2)	131/626 (20.9)	484/973 (49.7)	504/1874 (26.9)	72/295 (27.9)	39/646 (6.0)
Without prescription**	404/593 (68.1)	2/139 (1.4)	204/526 (38.8)	532/754 (70.6)	1/97 (1.0)	0/45 (0)

The number of households was adjusted for South Africa (600) and Mozambique (650) due to higher costs of field work. Respondents were the representatives of the surveyed households. *Respondents who answered “Yes” to the question “As far as you are aware, has anybody in this household taken antibiotics in the last month?”, N is the total number of households with a response to this question; **Courses of antibiotics that were obtained without a prescription.

Supplementary Table 7. Factors associated with purchasing WATCH-group antibiotics

Country	DDD supplied			Purchasing WATCH antibiotics		
	**Total DDD**	**Watch (DDD,%)**	**Access (DDD,%)**	**Crude OR (95% CI)**	**Adjusted OR (95% CI)**	**p value**
**Bangladesh**	**9,934**	**7,414 (74·6)**	**2,520 (25·4)**			
Without prescription	4,031 (40·6)	2859 (70·9)	1,172 (29·1)	1·28 (1·07 – 1·55)	1·26 (1·04 - 1·53)	0·02
For children	4,488 (45·2)	3226 (71·9)	1262 (28·1)	1·62 (1·34 – 1·96)	1·54 (1·25 - 1·90)	<0·001
**Mozambique**	**6,394**	**1,110 (17.4)**	**5,284 (82·6)**			
Without prescription	804 (12·6)	96 (12·0)	707 (88·0)	1·13 (0·64 - 1·98)	1·19 (0·67 - 2·1)	0·56
For children	934 (14·6)	26 (2·8)	908 (97·2)	0·13 (0·64 - 1·98)	0·14 (0·05 - 0·34)	<0·001
**Vietnam**	**7,222**	**3,414 (47·3)**	**3,664 (50·7)**			
Without prescription	3,687 (51·1)	1,830 (49.6)	1,837 (49.8)	0·81 (0·62 - 1·05)	2.62 (1·78 - 3·87)	<0·001
For children	1688 (23·4)	905 (53.6)	736 (43.6)	2·71 (2·07 - 3·56)	2·56 (1·84 - 3·55)	<0·001
**Ghana***	**7,105**	**2,026 (28.5)**	**5,072 (71.4)**			
Without prescription	1,768 (24·9)	402 (22·7)	1,366 (77·3)	1·65 (1·37 - 1·99)	1·73 (1·43 - 2·1)	<0·001
For children	2,061 (29·0)	334 (16·2)	1,727 (83·8)	0·58 (0·47 - 0·72)	0·57 (0·46 - 0·71)	<0·001
**Thailand**	**3,727**	**1,008 (27·0)**	**2,719 (73·0)**			
Without prescription	34 (1·6)	27 (79·3)	7 (20·7)	2·61 (0·32 – 21·43)	2·82 (0·33 - 24·03)	0·34
For children	853 (22·9)	542 (60·0)	311 (40·0)	2·36 (1·55 – 3·59)	2·73 (1·73 - 4·32)	<0.001
**South Africa**	**2,052**	**315 (15·4)**	**1764 (84·6)**			
Without prescription	33·4 (1·6)	0 (0)	33·4 (100)	1·03 (0·12 – 8·73)	0·72 (0·08 - 6·43)	0·77
For children	771 (37·6)	65 (8·4)	706 (91·6)	0·41 (0.21 – 0·78)	0·41 (0·2 - 0·82)	0·01

DDD: Defined Daily Dose. OR: odds ratio from multivariable logistic regression model to examine the factors associated with the purchasing Watch antibiotics, adjusted for age, sex, non-prescription, purchase for children and type of suppliers (public/private). Here we presented only two associated factors: purchasing without prescription and purchasing for children.

*Mean data of both sites was presented

Supplementary Table 8. Comparison between Kintampo and Dodowa in Ghana about antibiotic access and use

Characteristic	Kintampo	Dodowa	p value
**Density of antibiotic suppliers (n)**	134	110	
Non-licensed (n,%)	3 (2·2)	7 (6·4)	
Private (n,%)	95 (70·9)	101(91·8)	
**Care-seeking behavior**			
In mild illness (n)	*5399*	*4343*	
Drug store	**3097 (57·4)**	**1870 (43·1)**	
Private facility	660 (12·2)	318 (7·3)	
Public facility	1273 (23·6)	1533 (35·5)	
In severe illness	*5394*	*4352*	
Drug store	86 (1·6)	132 (3·0)	
Private facility	1074 (19.9)	640 (14·7)	
Public facility	**4109 (76·2)**	**2996 (68·8)**	
**Non-prescription purchasing (%)**	38·3 (861/2249)	34·1 (337/988)	
**Antibiotic consumption (DDD)**	8658	5564	
Access (DDD, %)	6471 (74·7)	3686 (66·2)	
Watch (DDD, %)	2179 (25·2)	1873 (33·7)	
Without prescription (OR, 95%CI)	3·83 (2·62-5·61)	5·02 (2·99-8·41)	< 0·001
For child patients	0·3 (0·19-0·46)	0·25 (0·14-0·47)	< 0·001

Within the same policy background and relatively equal density of suppliers, antibiotics access and use were mainly similar across two contexts (rural versus sub-urban), except higher frequency of household antibiotic use in Kintampo site which might be linked to higher burden of under-five mortality.

Supplementary Table 9. Family use of antibiotics in the previous month in the second survey round and associated factors in a multivariable generalized linear model including significant variables from univariable analysis

Variable*	Categories	OR (95%CI)	p value
Site**	Bangladesh	reference	-
	Mozambique	0.56 (0.43-0.74)	<0.0001
	Vietnam	1.22 (0.97-1.54)	0.08
	Ghana (Dodowa)	0.56 (0.42-0.75)	<0.0001
	Thailand	0.57 (0.41-0.78)	0.0005
	South Africa	0.20 (0.13-0.29)	<0.0001
Family use of antibiotics in the previous survey	No	reference	-
	Yes	1.38 (1.19-1.59)	<0.0001
Number of people in the household	1-4	reference	-
	>4	1.30 (1.13-1.49)	0.0003
Obtaining information about medicine from drugstores	No	reference	-
	Yes	1.21 (1.02-1.43)	0.03

* all variables in the table were included

** data from Kintampo could not be linked between round 1 and round 2 due to a lack of identified numbers.

Appendix. Health care delivery system and legal framework country scans by region

Asia

Bangladesh

Bangladesh has a pluralistic healthcare system, which is highly unregulated and consists mainly of four key actors: government, for-profit private sector, not-profit private sector (mainly the non- governmental organizations [NGOs]), and the international development organizations. The public healthcare services include four levels: community, primary, secondary, and tertiary level healthcare. The private sector also has health facilities ranging from individual doctors' offices to high-end tertiary level international standard hospitals. Public healthcare is highly subsidized by the government, with limited payments required from patients, especially for the outpatient care. Health insurance, both national and private, is very limited.

In Bangladesh, there are a number of legislations on drugs in general with main objectives are to ensure easy accessibility to essential drugs with affordable price, standard quality of drugs and rational use of drugs through appropriate prescribing and dispensing the health care professionals. The National Strategy for Antimicrobial Resistance Containment (ARC) has been established since 2012 to initiate implementation of integrated AMR activities through multi-sectoral approach which include promote and ensure rational use of antimicrobial agents in human health, livestock and fisheries. One of the activities taken by the AMRC is to draft National Antibiotic Policy on antimicrobials (AMs). According to National Drug Policy (NDP) 2016, retail sales of drugs (including antibiotics) is prohibited without prescription by registered physicians/veterinarians other than the over-the-counter drugs. However, lack of penalties for infringement with a focus on antibiotics make non-compliance is therefore common, particularly in private pharmacies.

Vietnam

Healthcare facilities in Vietnam are divided into four levels by administrative structure: central, provincial, district and commune. The healthcare system has a mixture of public and private sector. Private hospitals provide more than 60% of outpatient services and have become an important component of the national health system. A health insurance system was introduced

in 1993, and the government has made a considerable effort to achieve universal coverage, reaching 90% of the population by 2020. Since the market reforms in 1986 in Vietnam, 21,600 private pharmacies now exist, with easy access to medicines as a result. The private pharmacies also provide consulting for customers about drug use.

In Vietnam, the regulatory framework for antimicrobial use in human is stipulated in the Law on Pharmacy promulgated by the National Assembly in 2005 and updated in 2016. This law requires all antimicrobials to be dispensed only with a prescription. Despite regulations, most drug sellers continue to sell antimicrobials freely, without a prescription. Currently, there is no sanction for not complying with regulations regarding selling prescription-only drugs without a prescription.

In 2013, Vietnam became the first country in WHO's Western Pacific Region to approve a national action plan (NAP) to combat antimicrobial resistance. The plan was developed in response to the call from the World Health Organisation to have a timely plan to deal with antimicrobial resistance and based on data from a GARP situation analysis. Responsible use of antimicrobials in both humans and animal is one of the main expected outcomes of NAP by 2020.

Thailand

There are three levels of healthcare delivery in Thailand, namely, primary, secondary and tertiary. The primary care unit provides basic curative care, health promotion, and prevention. This level is exemplified by sub-district health promoting hospitals. Secondary care emphasizes curative care with various degrees of specialization, and is characterized by district, provincial, and regional hospitals. Finally, tertiary care outlets provide the most specialized services and include university hospitals and large private hospitals. The main actors in the formal healthcare delivery system in Thailand are both public and private. Public facilities cater to about three-fourths of the population. Private sector includes hospitals, polyclinics and clinics. In addition to the formal healthcare delivery system, there are numerous informal healthcare providers in Thailand. These include pharmacies, drugstores, grocery stores and other alternative care providers such as traditional healers.

In Thailand, antibiotics (ABs) are classified by Thailand's Drug Act as potentially dangerous drugs. There is law (National Drug Act), smart use program and national strategic plan govern the

distribution of categorised antibiotics. Only drug store for modern medicine type one which can be dispensed by all authorised health facilities can dispense Abs by pharmacist without a prescription. Licensed physicians, pharmacists and veterinaries can dispense antibiotics for their own customers/patients. An 'Antibiotics Smart Use Program' was started in 2007 to reduce unnecessary prescriptions of antibiotics for common self-limiting conditions. Lastly, National Strategic Plan on AMR has been implemented since 2017 up to 21 with six strategies including regulation of antimicrobial distribution and appropriate use of antimicrobials with governance mechanisms to implement and sustain actions.

Africa

Ghana

In Ghana, most health care is provided by the government and is largely administered by the Ministry of Health and Ghana Health Services. The healthcare system has five levels of providers: health posts, health centers and clinics, district hospitals, regional hospitals and tertiary hospitals. Health posts are the first level of primary care for rural areas. Health care is very variable through Ghana. Urban centres are well served, and contain most hospitals (both self-financed private (SFP) and government (Ghana Health Service) providers, clinics, and pharmacies in the country whereas Christian Health Association of Ghana (CHAG) providers and chemical sellers are concentrated in rural and urban-poor areas. Chemical sellers, by their number and geographical location, appear to represent the greatest and most accessible source of services in rural and urban-poor areas.

In Ghana, most antibiotics can only be dispensed by a qualified Medical Officer and Pharmacist. Failure to comply may lead to revoke of license or a penalty not exceeding about USD 112 on a license holder. However, it is challenging to fully enforce regulations that limits over-the-counter dispensing of antibiotics. The national action plan (NAP) is a synthesis of the steps needed to help Ghana achieve her aspirations of combating the AMR phenomenon in country based on the "One Health" concept. The "One Health" concept emphasized that human health and animal health are interdependent and bound to the health of the ecosystems in which they exist[31]. The NAP defines the role of all implementing stakeholders and shall be the focus for investments into AMR

containment efforts in Ghana. Currently, the National Policy on Antimicrobial Use and Resistance and NAP has been launched and full implementation was scheduled in the period of 2018-2021.

Mozambique

The public healthcare system in Mozambique is basic and limited. Public hospitals are subject to frequent staff and supply shortages, and most have excessively long waiting times for even basic care. Some rural areas have little to no public healthcare options, with locals often travelling for hours to get to the nearest government clinic. Private healthcare is only available in large cities and consumed by high-income groups. At the time of the study there were 845 retail pharmacies operational throughout Mozambique. Poor access to primary health care facilities has made it difficult for government efforts to deal effectively with the health situation. According to the Mozambique Poverty Reduction Strategic Program (PARPAII), only 36% of people have access to a health facility within 30 minutes of their homes. About 30% of the population are not able to access health services and only 50% have access to an acceptable level of health care.

In Mozambique, there are no specific policies regarding access and antibiotics usage. Nonetheless, there is a global medicine law in which antibiotics are included. It's stated that "Medicines requiring prescription- all antibiotics and any medicines labelled *'selling upon prescription'* should not be dispensed without the prescription". Government is currently making great effort to consolidate the pharmaceutical sector. In order to ascertain the extent of antibiotic resistance and use in the country, the Ministry of Health jointly with a Multidisciplinary working group, Global Antibiotic Resistance Partnership (GARP-Mozambique), conducted a "situation analysis and recommendation antibiotic use and Resistance in Mozambique" since August 2012. The same group developed the NAP, who received technical approval from Ministry of Health. The NAP was approved by the Ministry of Health and the Ministry of Agriculture and Food Security in 2019.

South Africa

In South Africa, the public healthcare system is structured in different levels including: primary health care (including mobile and satellite clinics) which is running by nurses provides common

health needs; community health centers are larger clinics and usually have doctors; and hospitals for emergency treatment and serious illnesses. About 71% of the population uses public sector while the private sector serves around 27% and mainly available in urban areas. In South Africa, the department of health has created a strategy framework, the Antimicrobial Resistance National Strategy Framework 2014- 2024,[32] that is largely informed by the World Health Organisation resolution on Combating Antimicrobial Resistance Including Antibiotic Resistance. The main aim of the framework is to control antibiotic resistance of bacterial infections, other than tuberculosis (TB) as TB frameworks and strategies have been created, enforced and operational for some time. The framework is underpinned by four strategic objectives which include the promotion of appropriate use of antimicrobials in human and animal health. Education of both healthcare and community members was identified as a key strategic enabler.

Antibiotic legal framework across sites

Site	Prescription regulation	National Action Plan
Bangladesh	Prohibited (National Drug Policy, 2016)<br>Lack of penalties	National Strategy for AMR Containment (ARC) 2012
Mozambique	International regulations	NAP (by GAPR-Mozambique) is still waiting for approval
Vietnam	Drug Law 2005<br>Updated 2016	NAP 2013
Ghana	Dispensed by licensed HCWs including pharmacists<br>Penalties	NAP has been launched and fully implemented in 2019
Thailand	National Drug Act	Antibiotics Smart Use Program 2007<br>National Strategic Plan on AMR 2017
South Africa	Prescription only<br>International regulation	AMR National Strategy Framework 2014-2024
